# Combination Therapy with Atorvastatin and Amlodipine Suppresses Angiotensin II-Induced Aortic Aneurysm Formation

**DOI:** 10.1371/journal.pone.0072558

**Published:** 2013-08-13

**Authors:** Kikuyo Takahashi, Yasuharu Matsumoto, Zhulanqiqige Do.e, Masanori Kanazawa, Kimio Satoh, Takuya Shimizu, Akira Sato, Yoshihiro Fukumoto, Hiroaki Shimokawa

**Affiliations:** 1 Department of Cardiovascular Medicine, Tohoku University Graduate School of Medicine, Sendai, Japan; 2 Department of Transplantation, Reconstruction and Endoscopic Surgery, Tohoku University Graduate School of Medicine, Sendai, Japan; University Medical Center Utrecht, Netherlands

## Abstract

**Background:**

Abdominal aortic aneurysm (AAA) is a life-threatening vascular disease. It is controversial whether statin and calcium channel blockers (CCBs) has an inhibitory effect on the expansion of AAA. Some studies reported that CCBs have an inhibitory effect on Rho-kinase activity. Rho-kinase plays an important role in the pathogenesis of various cardiovascular diseases. However, there is no study reporting of the association between Rho-kinase and human AAAs.

**Methods and Results:**

Experimental AAA was induced in Apolipoprotein E-deficient (ApoE^-/-^) mice infused with angiotensin II (AngII) for 28 days. They were randomly divided into the following 5 groups; saline infusion alone (sham), AngII infusion alone, AngII infusion plus atorvastatin (10 mg/kg/day), AngII infusion plus amlodipine (1 mg/kg/day), and AngII infusion plus combination therapy with atorvastatin (10 mg/kg/day) and amlodipine (1 mg/kg/day). The combination therapy significantly suppressed AngII-induced increase in maximal aortic diameter as compared with sham, whereas each monotherapy had no inhibitory effects. The combination therapy significantly reduced AngII-induced apoptosis and elastin degradation at the AAA lesion, whereas each monotherapy did not. Moreover, Rho-kinase activity, as evaluated by the extent of phosphorylation of myosin-binding subunit (a substrate of Rho-kinase) and matrix metalloproteinase activity were significantly increased in the AngII-induced AAA lesion as compared with sham, both of which were again significantly suppressed by the combination therapy. In human aortic samples, immunohistochemistory revealed that the activity and expression of Rho-kinase was up-regulated in AAA lesion as compared with abdominal aorta from control subjects.

**Conclusions:**

Rho-kinase is up-regulated in the aortic wall of human AAA. The combination therapy with amlodipine and Atorvastatin, but not each monotherapy, suppresses AngII-induced AAA formation in mice in vivo, for which Rho-kinase inhibition may be involved.

## Introduction

Abdominal aortic aneurysm (AAA) is a substantial burden in the developed countries, due in part to aging of the society [1]. However, no effective pharmacological strategies are established to suppress the development of AAA despite of the introduction of improved screening and diagnostic tools [2].

Recent studies demonstrated that both 3-hydroxy-3-methyl-glutaryl coenzyme A reductase inhibitors (statins) and calcium channel blockers (CCBs) exert beneficial effects on cardiovascular disease [3–5]. It is, however, controversial whether statins suppress the development and progression of AAA [6–8]. Further, it was newly reported that CCBs enhance sac shrinkage after endovascular aneurysm repair in humans [9] and prevent AAA formation in experimental study [10].

The pathological features of AAA include chronic inflammation [11–13], oxidative stress [14,15] and activation of proteolytic enzymes that lead to degradation of the elastic media and the development of this disorder, although the underlying mechanisms remain largely unknown [16]. We have previously demonstrated that Rho-kinase plays an important role in the pathogenesis of atherosclerotic vascular disease [17]. However, activity or expression of Rho-kinase at the AAA lesion remains to be elucidated. Recently, pleiotropic effects of statins and CCBs through the Rho-kinase pathway have been suggested in animals and humans [18–20].

Thus, we hypothesized that combination therapy with statins and CCBs may synergistically exert pleiotropic effects, such as anti-inflammatory effects associated with Rho-kinase pathway inhibition, resulting in the suppression of AAA growth. In the present study, we examined whether 1) statins, calcium channel blockers, or their combination therapy suppress AAA formation in mice model, 2) Rho-kinase is involved in the mechanism of AAA inhibition by pharmacological therapy and 3) Rho-kinase is up-regulated in the human AAA.

## Materials and Methods

### Ethics Statement

Animal care and the experimental procedures were approved by the Guidelines on Animal Experiments of Tohoku University and the Japanese Government Animal Protection and Management Law (No. 105-2011). All animal experiments were performed in accordance with the Guide for the Care and Use of Laboratory Animals published by the U.S. National Institute of Health (NIH Publication). We retrospectively conducted all protocol reusing human stored samples only after approval by the ethics committee of Tohoku University Graduate School of Medicine (No. 2012-1-300). All experiments using human tissue were performed in accordance with the principles outlined in the Declaration of Helsinki.

### Animals

Male apolipoprotein E-deficient (ApoE^-/-^) mice on a C57BL/6 background were obtained from Kyudo (Saga, Japan) and were bred in a pathogen-free environment. All mice were maintained on a normal mouse chow diet.

### Drugs

**Figure 1:**
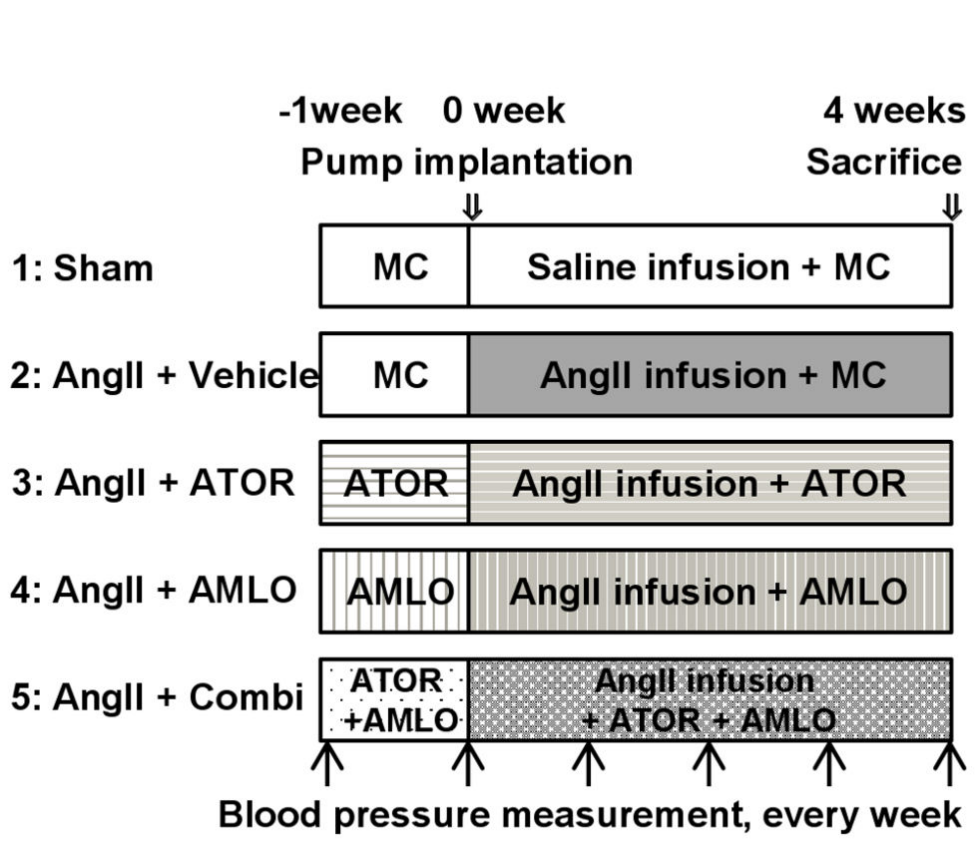
Experimental study design. In the present study, 5 treatment groups were made. Group 1 was sham; group 2, angiotensin II plus vehicle; group 3, angiotensin II plus atorvastatin; group 4, angiotensin II plus amlodipine; group 5, angiotensin II plus combination of atorvastatin and amlodipine. MC, methylcellurose; AngII, angiotensin II; ATOR, atorvastatin; AMLO, amlodipine; Combi, combination of atorvastatin and amlodipine.

A total of 182 ApoE^-/-^ mice at 6 months of age were randomly divided into the following 5 experimental groups; group 1, saline infusion with 0.5% methylcellulose treatment (sham); Group 2, angiotensin-II (AngII) infusion with methylcellulose treatment (AngII); group 3, AngII infusion plus amlodipine (1 mg/kg/day) (AMLO); group 4, AngII infusion plus atorvastatin (10 mg/kg/day) (ATOR); and group 5, AngII infusion plus combination therapy with amlodipine (1 mg/kg/day) and atorvastatin (10 mg/kg/day) (Combi) (Figure 1). Each atorvastatin (Pfizer, Groton, CT, USA) or amlodipine (Pfizer) was suspended in 0.5% methylcellulose and administrated by gavage once a day. After 7 days treatment with each drug by gavage, Alzet osmotic minipumps (Model 2004, Durect Corporation, Cupertino, CA, USA) were implanted into the mice to continuously deliver AngII subcutaneously at a dose of 2,500 ng/kg/min or saline vehicle for 28 days, as previously described [21]. For the pump implantation, the mice were anaesthetized with isoflurane for 3-5 min using an automatic delivery system (Univentor 400 Anaesthesia Unit, Univentor, Zejtun, Malta) that provides a steady concentration of 1.8% isoflurane. We confirmed loss of reflex by pricking the tail of mice with a needle and implanted a pump under continuous anaesthesia with 1.8% isoflurane.

### Blood pressure measurements

Systolic blood pressure was measured at room temperature by a tail cuff method using an MK2000 blood pressure monitor (Muromachi Kikai, Tokyo, Japan), under conscious conditions. Mice were acclimated to the procedures of blood pressure measurement for a week preceding actual data collection. Systolic blood pressures were measured 8-days before pump implantation to record baseline blood pressures, 1-day before pump implantation, and repeated weekly during AngII infusion.

### Histology and immunohistochemistry

Morphological and immunohistochemical analyses were performed as previously described [22]. For morphometric studies, the mice were anaesthetized with 3.0% isoflurane followed by blood collection and perfusion via cardiac puncture with PBS and subsequently 10% formalin at physiological rate with infusion pump (Harvard apparatus, Holiston, MA, USA). The depth of general anaesthesia was assessed by pinching tail of the mouse. Any reaction from the mouse indicated that the anaesthesia was too light and that higher concentration of isoflurane should be given. The entire aorta from the ascending aorta to the iliac bifurcation were exposed and placed in 10% formalin for 24 h to complete the fixation. Abdominal aortic diameters were quantified by measuring the maximal diameter of fixed aortic tissues using ImageJ (NIH) Software. Fixed aortic tissues were then embedded in paraffin, and then cut into 3-µm sections for morphological and immunohistochemical analysis. Immunostaining was performed using rabbit anti-mouse F4/80 (Abcam, Cambridge, UK), CD3 (Abcam), ROCK1 (Abcam), ROCK2 (Abcam), cyclophilin A (CyPA, Abcam), and α-actin (Thermo, Fisher Scientific, Fremont, CA, USA). The numbers of F4/80-positive cells and CD3-positive cells were determined by counting cells in a blind manner at x400, respectively. The average number from 7 fields was used as an individual value.

To localize cells undergoing nuclear DNA fragmentation, in situ terminal deoxynucleotidyl transferase-mediated dUTP nick-end labeling (TUNEL) was performed using in situ apoptosis detection kit (Roche Biochemicals, Mannheim, Germany) according to manufacturer’s protocol. Both positive and negative control slides were processed at the same time in each experiment. The presence of apoptotic cells was scored as described previously [23]; grade 0, absent; grade 1 ,mild (<25%); grade 2, moderate (>25%, <50%); grade 3, severe (>50%). Each aortic tissue was scored in 7 fields and the average was used as an individual value.

### Gelatin zymography

For analysis of MMPs activity in the aortic tissues, gelatin zymography was performed as previously described [24]. After homogenization and protein quantification, equal volumes of aortic tissue extract (10-μg protein) were separated by electrophoresis on 10% SDS-polyacrylamide gels which contained 0.1% gelatin (Novex EC 61752, Life Technologies, Carlsbad, CA, USA). After re-natured, they were incubated with developing buffer (Novex Zymogram developing buffer LC2671, Life Technologies) at 37° C for 48 hours with gentle agitation. The gels were stained with Coomassie Brilliant Blue, and gelatinolytic activity was quantified. The sum of MMP-2 and pro-MMP-2 bands was measured as total MMP-2 activity [25].

### Western blotting

Isolated abdominal aortic tissues were homogenized and proteins were extracted. The same amount of extracted protein (10-μg) was loaded for SDS-PAGE immunoblot analysis and transferred to polyvinylidene difluoride (PVDF) membranes. The membranes were blocked with 10% non-fat milk in Tris buffer solution containing 0.1% Tween-20 for one hour at room temperature with gentle agitation. The membrane was immunoblotted with anti-myosin binding subunit (MBS) (Millipore, Billerica, MA, USA), rabbit anti-phospho-MBS antibody (Millipore), and mouse anti-α-actin antibody (Abcam). After incubating with HRP-conjugated anti-mouse (Sigma Aldrich, St. Louis, MO, USA) or anti-rabbit IgG antibody (Cell Signaling, Danvers, MA, USA), blots were visualized by the enhanced chemiluminescence system (ECL Western Blotting Detection kit, GE healthcare). Each band was normalized by corresponding value of β-actin as an internal control. Densitometric analysis was performed by ImageJ (NIH) Software. To quantify the activity of Rho-kinase, the ratio of phosphorylated-MBS to MBS was calculated.

### Human samples

We retrospectively reused the stored samples that had been obtained during surgical repair of AAA from the patients showing dilatation of abdominal aorta with a diameter >5 cm (n=6) as well as the autopsy specimens from patients who died of unrelated causes served as control (n=3). Paraffin-embedded cross-sections of abdominal aorta (3-μm) were stained with hematoxylin-eosin (HE). Immunohistochemical staining was performed as well on paraffin-embedded tissue sections using rabbit anti-human ROCK1 (Abcam), ROCK2 (Abcam), phospho-MBS (Abcam), α-actin (Thermo Fishier Scientific) antibodies.

### Statistical analysis

Samples were coded so that analysis was performed without knowledge of which treatment had received. All values are expressed as mean±SEM. Chi-square test was performed for the analysis of mortality due to aortic rupture. The other statistical significance of differences among multiple groups was determined using ANOVA and post-hoc analysis with the Turkey’s test. Statistical significance was defined at P<0.05. 

## Results

### Blood pressure and lipid profile in AngII-induced AAA model in mice

After starting AngII infusion in mice, blood pressure was significantly and equally elevated in all of the AngII-infused groups as compared with saline-infused group (sham) (Table S1). Amlodipine (1 mg/kg/day) had no inhibitory effect on the blood pressure elevation in the present study. Similarly, all ApoE^-/-^ mice showed hyperlipidemia and there was no significant difference in the lipid profiles among the groups (Figure S1).

### Macroscopic and microscopic features of AAA in mice

**Figure 2:**
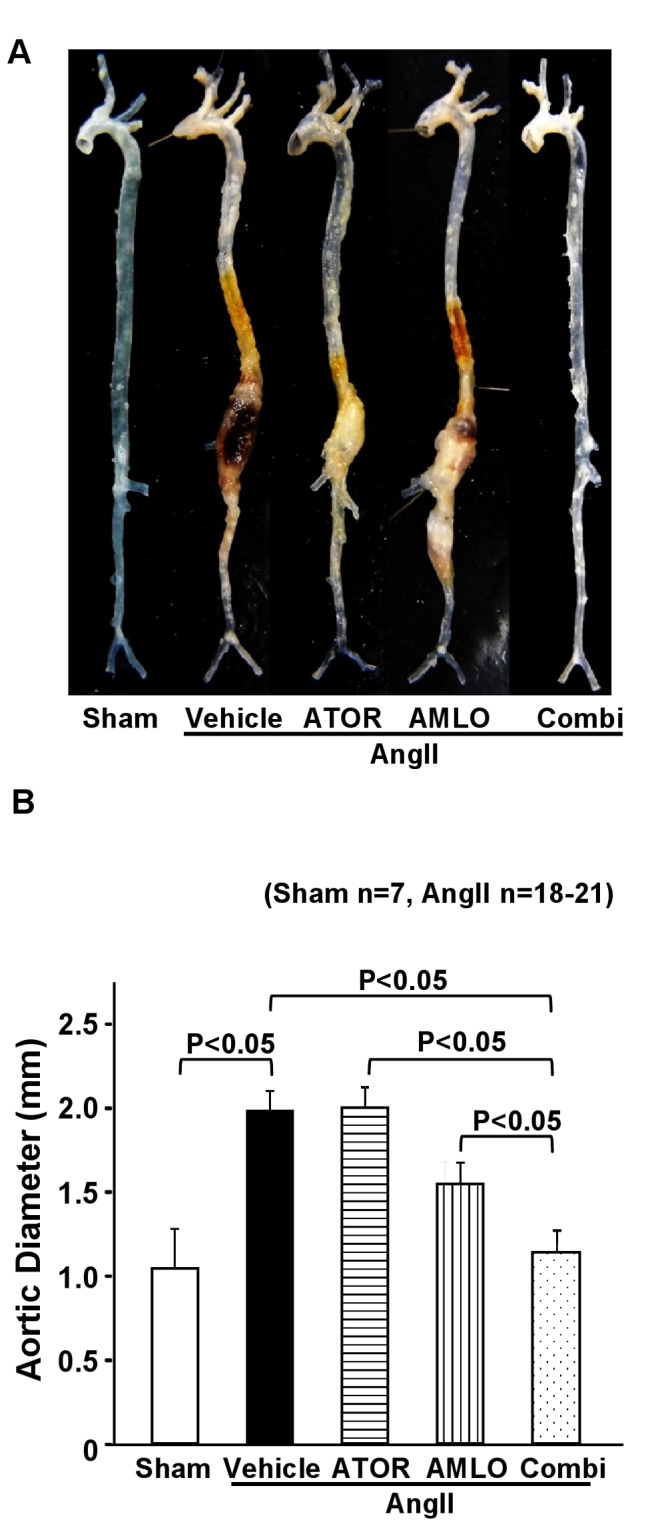
Combination therapy with atorvastatin and amlodipine suppresses AngII-induced AAA formation in ApoE^-/-^ mice. **A**. Representative photographs showing the macroscopic features of AngII-induced abdominal aortic aneurysm (AAA). **B**. Maximal abdominal aortic diameter in ApoE^-/-^ mice after AngII infusion for 4 weeks (sham, n=7; AngII, n=20; ATOR, n=21; AMLO, n=18; Combi, n=20). AngII, angiotensin II; ATOR, atorvastatin; AMLO, amlodipine; Combi, combination of atorvastatin and amlodipine. All values are expressed as mean±SEM.

No AAA formation was noted in the sham group (Figure 2A). The combination therapy with atorvastatin and amlodipine significantly suppressed AngII-induced increase in maximal aortic diameter [sham, 1.03±0.02 mm; AngII, 1.97±0.17 mm; ATOR, 1.98±0.19 mm; AMLO, 1.74±0.20 mm; Combi, 1.13±0.02 mm; P<0.01, AngII vs. Combi] (Figure 2B). Elastica-Masson staining demonstrated that the elastic lamellae were severely destroyed in the AngII group compared with the sham group, which was significantly suppressed in the combination group, but not in each monotherapy group (Figure S2, A-K). No mice in the sham group died. There was no significant difference in the mortality due to aortic rupture as determined by necropsy [AngII, 11.3% (5/44); ATOR, 5.1% (2/39); AMLO, 5.4% (2/37); Combi, 5.0% (2/40)] (Table S2).

### Vascular inflammation in mice

**Figure 3:**
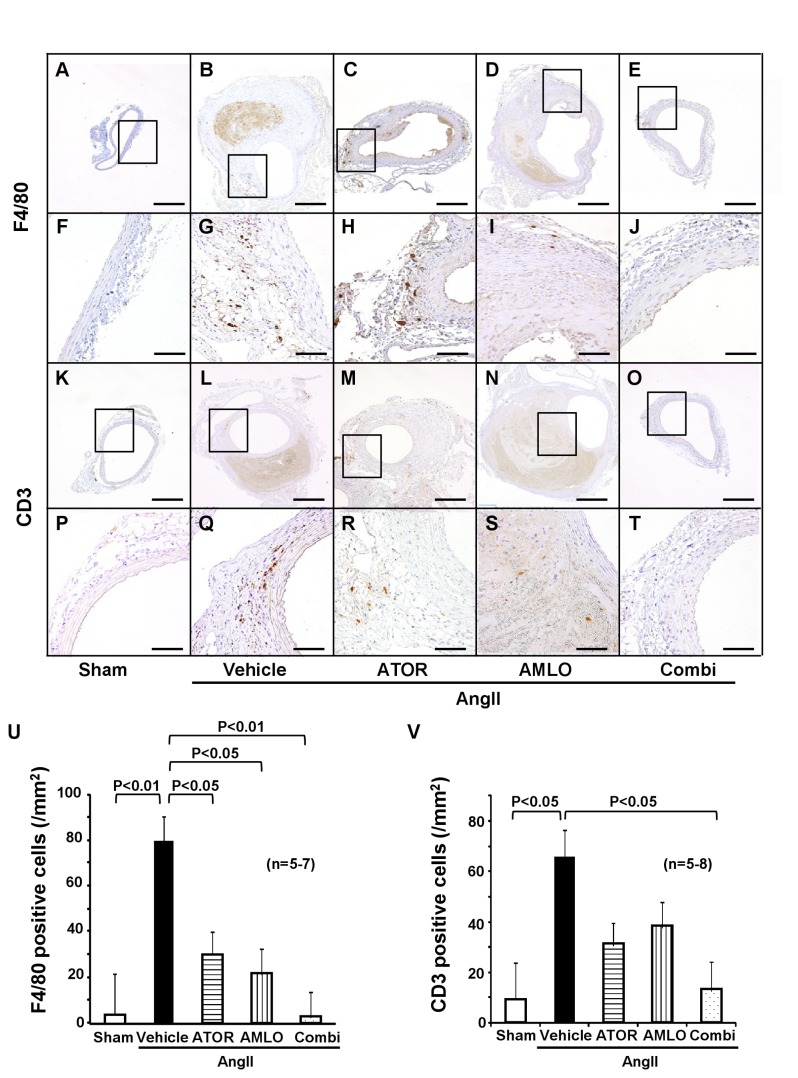
Combination therapy suppresses inflammatory cells infiltration at the AngII-induced abdominal aortic aneurysm in ApoE^-/-^ mice. **A**–**J**, Immunostaining for F4/80, a specific marker for mature macrophages. **K**–**T**, CD3, pan-leukocyte marker. **U**. Quantitative analysis of F4/80 positive cells (sham, n=5; AngII, n=7; ATOR, n=7; AMLO, n=7; Combi, n=7) and **V**. CD3 positive cells (sham, n=5; AngII, n=8; ATOR, n=8; AMLO, n=8; Combi, n=8). Scale bars indicate 500 µm (**A**–**E** and **K**–**O**) and 100 µm (**F**–**J** and **P**–**T**). AngII, angiotensin II; ATOR, atorvastatin; AMLO, amlodipine; Combi, combination of atorvastatin and amlodipine. Results are expressed as mean±SEM.

We evaluated the extent of infiltration of inflammatory cells. F4/80-positive macrophages and CD3-positive T-lymphocytes abundantly infiltrated to the adventitia and media of the aorta in the AngII group (Figure 3, A-T). Quantitative analysis showed that macrophage infiltration was significantly increased at the AAA lesion in the AngII group than in the sham group (Figure 3 U). F4/80-positive cells were located in the media and adventitia of aortic tissues. In contrast, the combination therapy significantly suppressed macrophage accumulation to the level in the sham group (P<0.01), while the monotherapy with atorvastatin also significantly reduced it as compared with the AngII group (P<0.05). T-lymphocyte infiltration also was significantly increased at the AAA lesion in the AngII group than in the sham group, whereas the combination therapy significantly suppressed it as compared with the AngII group (Figure 3 V). Ultrasonography showed apparent turbulent flow in the AAA lesion (Figure S3 A). Thus, we assessed endothelial shear stress using Krüppel-like factor2 (KLF2) as a factor that regulates vascular inflammation [26]. Expression of endothelial KLF2 was observed in the sham group and was down-regulated at the AngII-induced AAA lesion, which was restored in the combination group but not in each monotherapy group (Figure S3 B).

### Apoptosis in mice

**Figure 4:**
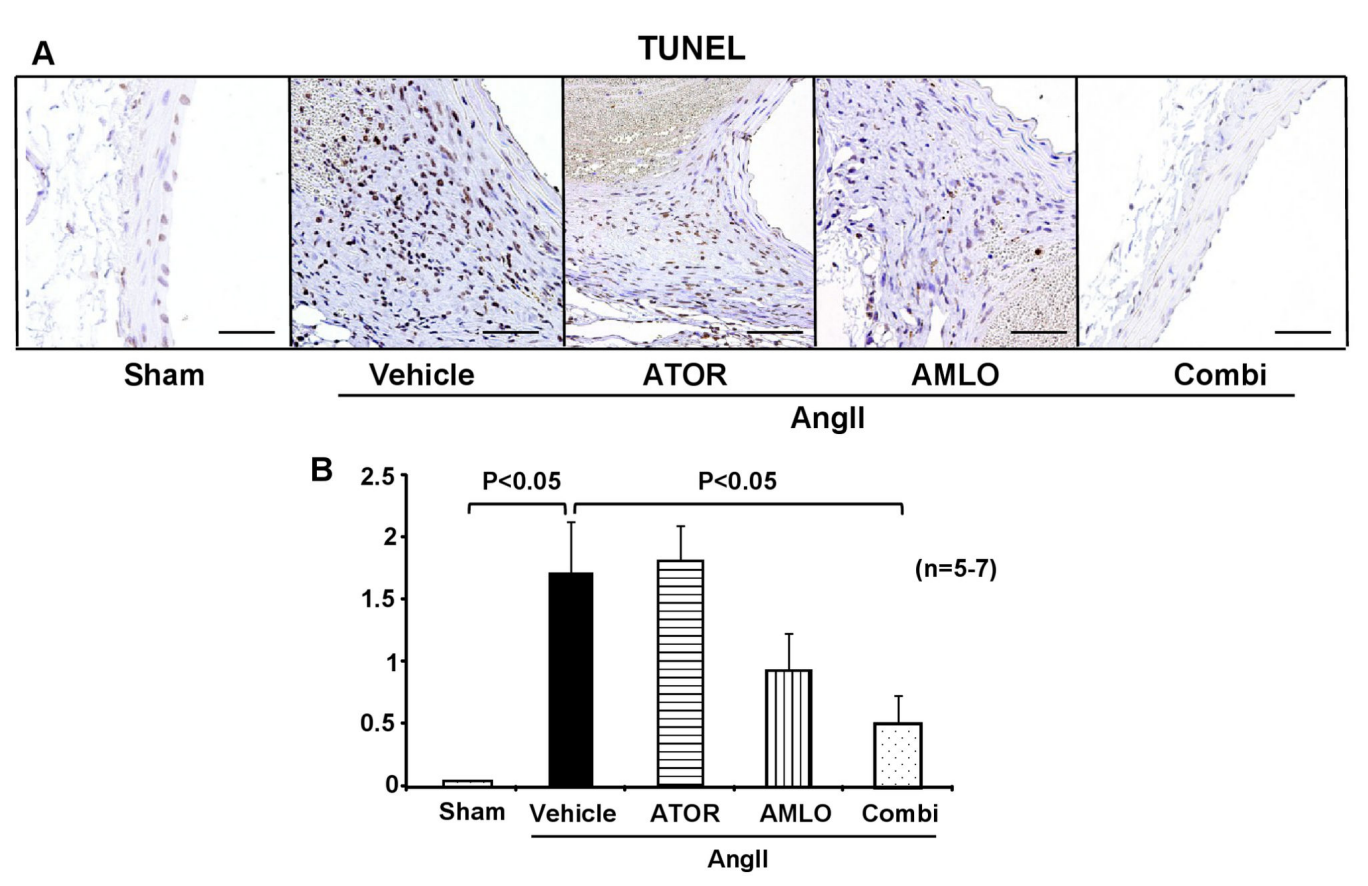
Combination therapy with atorvastatin and amlodipine suppresses apoptosis in ApoE^-/-^ mice. **A**. Representative photographs of terminal deoxynucleotidyl transferase dUTP nick end labeling (TUNEL) staining in ApoE^-/-^ mice after AngII infusion for 4 weeks. Scale bars indicate 100 µm. **B**. Semi-quantitative analysis of TUNEL-positive cells (sham, n=5; AngII, n=7; ATOR, n=7; AMLO, n=7; Combi, n=7). AngII, angiotensin II; ATOR, atorvastatin; AMLO, amlodipine; Combi, combination of atorvastatin and amlodipine. All values are expressed as mean±SEM.

Apoptosis is another important pathological mechanism that alters tissue structure in AAA. TUNEL-positive apoptotic cells in the media and adventitia were significantly increased in the AngII group than in the sham group. This increase in apoptosis induced by AngII was significantly suppressed in the combination group, but not in each monotherapy group (Figure 4).

### MMP activity in mice

**Figure 5:**
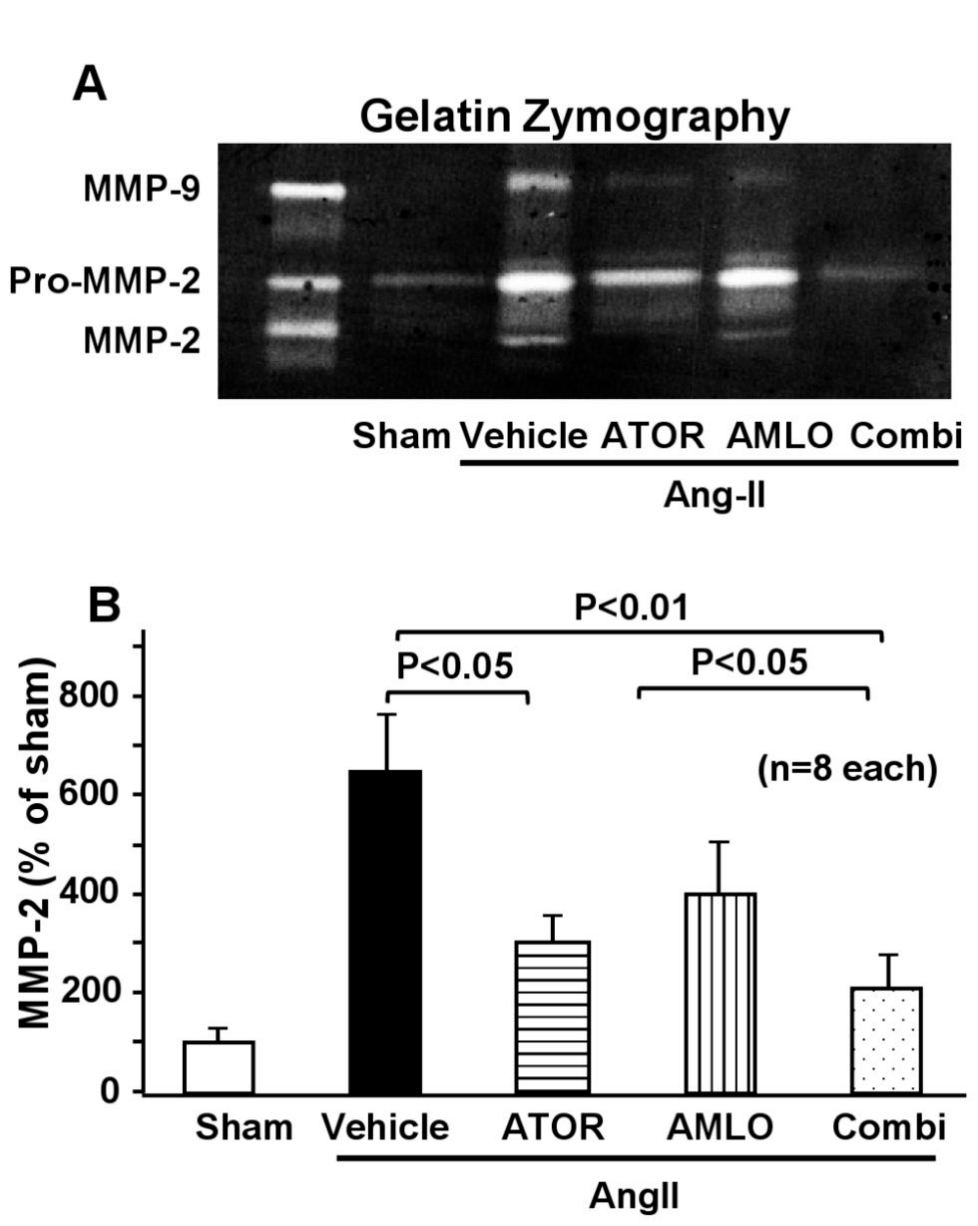
Effect of combined therapy with atorvastatin and amlodipine and each monotherapy on the matrix metalloproteinase activity in the abdominal aortic aneurysm. **A**. Representative gelatin zymogram. **B**. Quantitative analysis for MMP-2 activity. MMP, matrix metalloproteinase; AngII, angiotensin II; ATOR, atorvastatin; AMLO, amlodipine; Combi, combination of atorvastatin and amlodipine. Results are expressed as mean±SEM (n=8 each).

MMP-2 activity was significantly increased in the AngII group than in the sham group. Enhanced MMP-2 activity induced by AngII was significantly suppressed in the combination group, but not in each monotherapy group (Figure 5).

### Rho-kinase activity in mice

**Figure 6:**
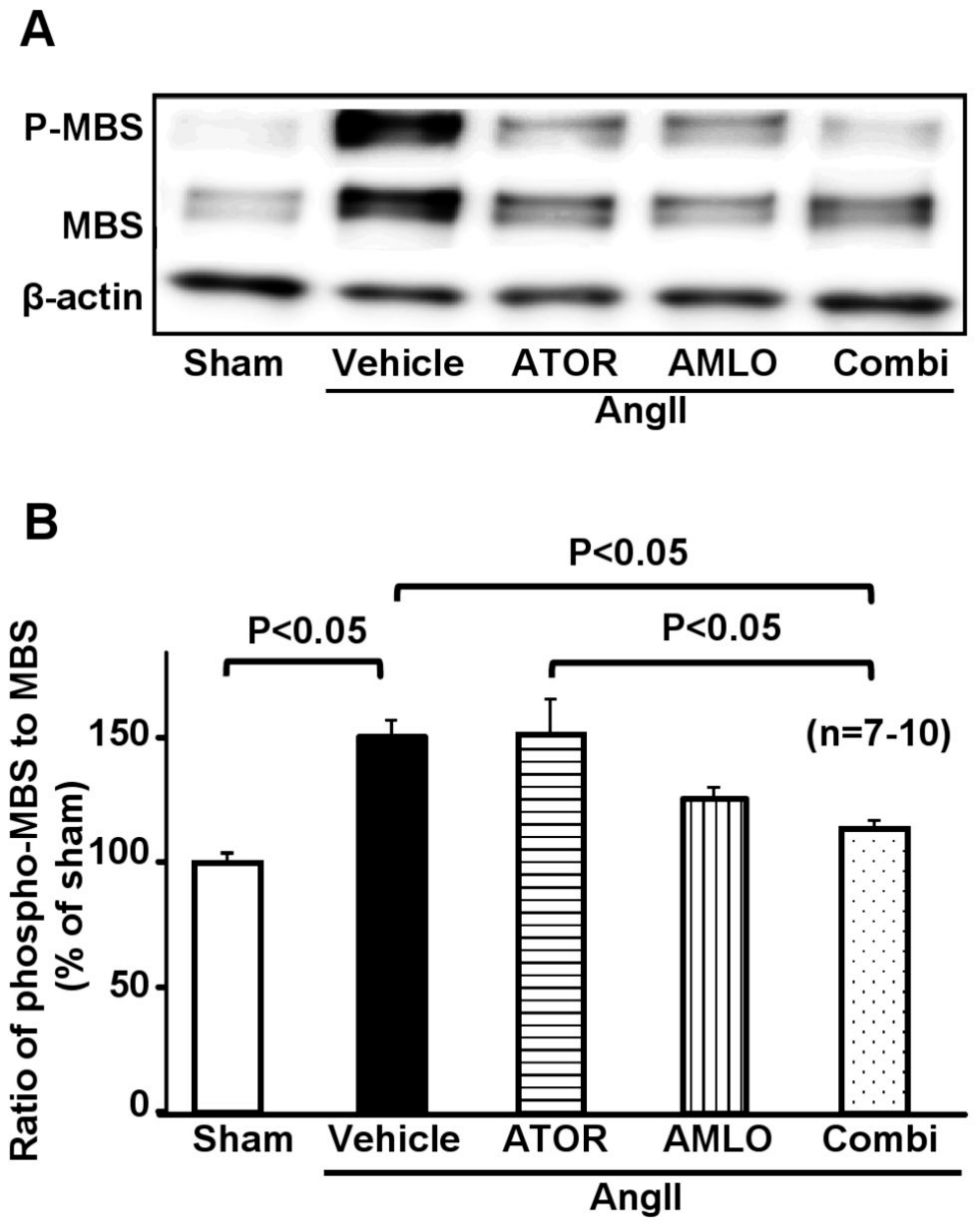
Combination therapy with atorvastatin and amlodipine suppresses Rho-kinase activation at AngII-induced AAA lesions in ApoE^-/-^ mice. **A**. Western blot analysis for Rho-kinase activity as expressed with the ratio of phosphorylated myosin binding subunit (pMBS) per MBS. **B**. Quantitative analysis of Rho-kinase activity (sham, n=7; AngII, n=10; ATOR, n=8; AMLO, n=7; Combi, n=9). AAA, abdominal aortic aneurysm; AngII, angiotensin II; ATOR, atorvastatin; AMLO, amlodipine; Combi, combination of atorvastatin and amlodipine. All values are expressed as mean±SEM.

We have previously demonstrated that Rho-kinase mediates AngII-induced vascular inflammation and remodeling [17]. However, Rho-kinase activity at the AAA lesion remains to be examined. Thus, we quantitatively evaluated the extent of MBS phosphorylation as a marker of Rho-kinase activity by Western blot analysis [27]. The ratio of phosphorylated-MBS to MBS was significantly increased at the AAA lesion in the AngII group as compared with the sham group, which was significantly suppressed in the combination group, but not in each monotherapy group (Figure 6).

### Rho-kinase expression and activity in mice

**Figure 7:**
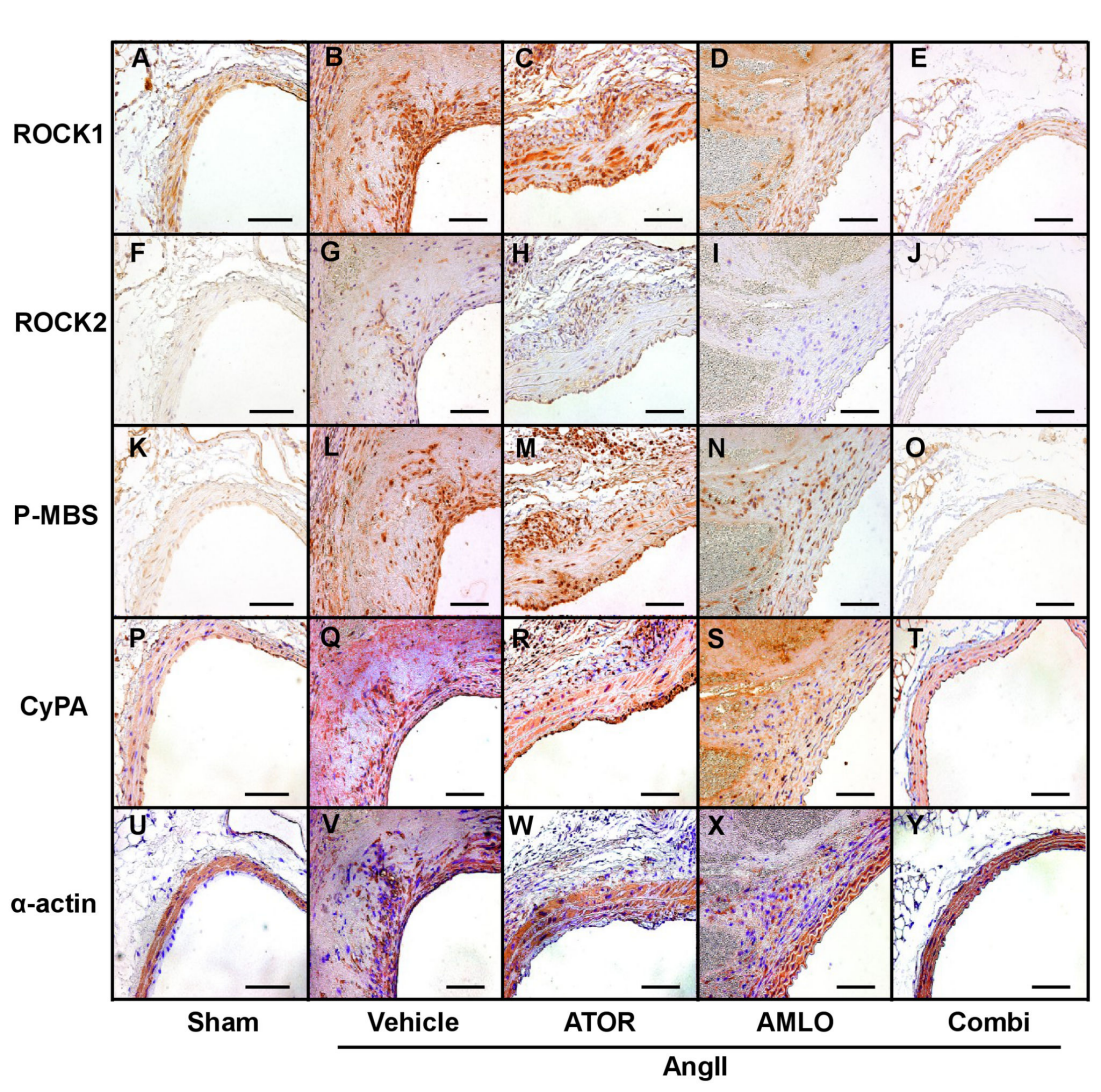
Combination therapy with atorvastatin and amlodipine suppresses Rho-kinase expression and activity and cyclophilin A expression in ApoE^-/-^ mice. Immunohistochemistry for ROCK1, ROCK2, phospho-MBS, CyPA, α-actin. Scale bars indicate 100 µm. AngII, angiotensin II; ATOR, atorvastatin; AMLO, amlodipine; Combi, combination of atorvastatin and amlodipine; MBS, myosin binding subunit; CyPA, cyclophilin A.

We examined Rho-kinase expression and activity at the AAA lesion. There are 2 isoforms of Rho-kinase, ROCK1 and ROCK2 [17]. Expression of Rho-kinase (ROCK1/2) and its activity (phospho-MBS) was also increased at the AAA lesion, including α-actin-positive smooth muscle cells in the AngII group than in the sham group (Figure 7, A-O). The combination therapy reduced both ROCK1 and ROCK2 expression induced by AngII, while atorvastatin monotherapy did not. Interestingly, amlodipine monotherapy seems to substantially reduce ROCK2 expression to the level in the combination therapy (Figure 7, F-J). We also examined expression of CyPA, an important regulator of Rho-kinase and AAA formation [28]. Expression of CyPA was also increased at the AAA lesion in the AngII group than in the sham group. Enhanced expression of CyPA induced by AngII was also decreased in the combination therapy but not in each monotherapy, in conjunction with expression and activity of Rho-kinase (Figure 7, P-T).

### Rho-kinase expression and activity in human AAA tissue

**Figure 8:**
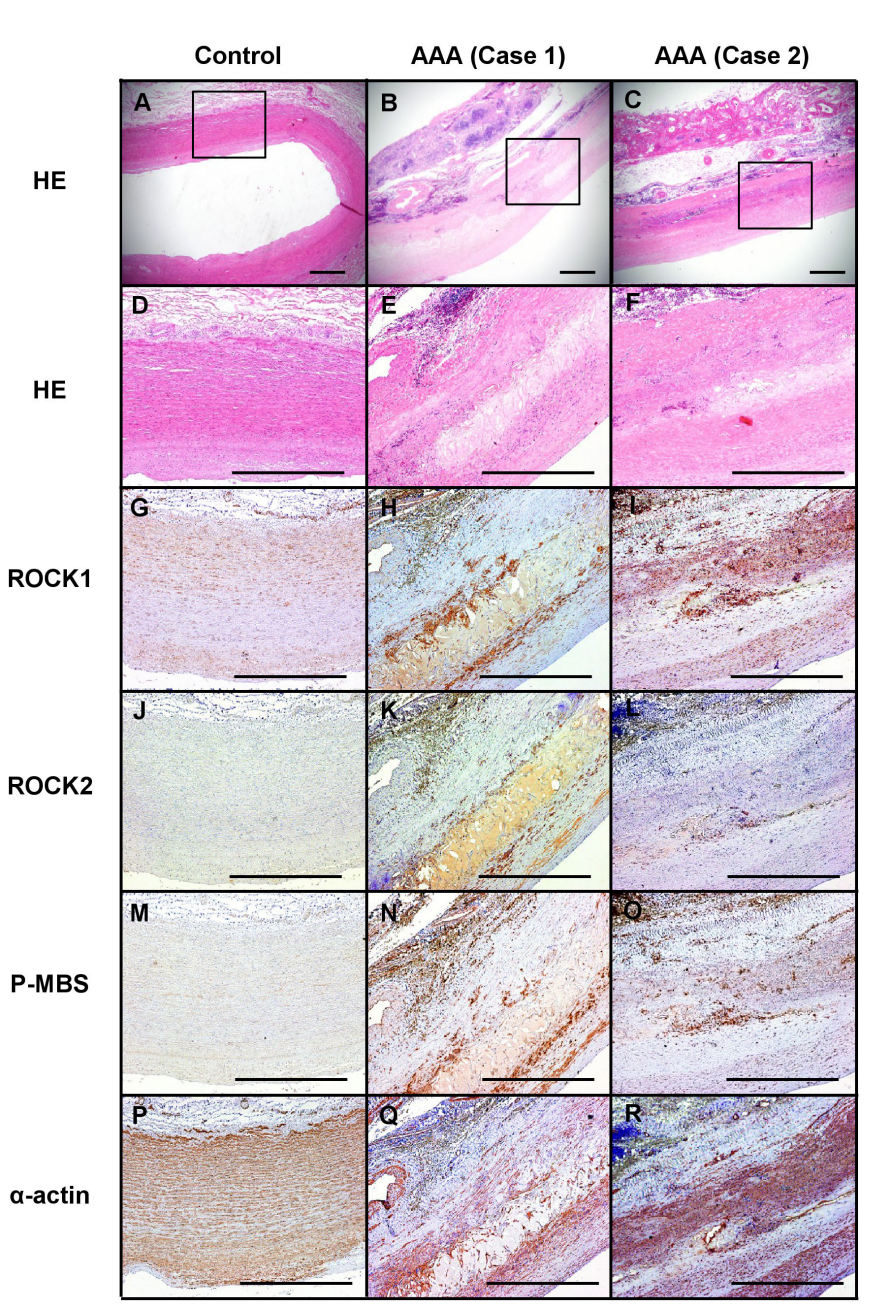
Enhanced Rho-kinase expression and its activity in the human AAA. **A**–**C**. Representative photographs of Hematoxylin-Eosin (HE) staining. **D**–**F**. High-power field of A-C (HE staining). **G**–**I**. Immunohistochemistry for ROCK1. **J**–**L**. ROCK2. **M-O**. Phosphorylated myosin binding subunit (pMBS). **P**–**R**. α-actin. Scale bars indicate 1.00 mm. AAA, abdominal aortic aneurysm.

We performed immunostaining of ROCK1, ROCK2 and phosphorylated MBS, a marker of Rho-kinase activity [17], in the abdominal aorta from the patients with AAA and control subjects without AAA (Figure 8). Expression of ROCK1 and ROCK2 was enhanced in the AAA lesion as compared with control (Figure 8, G-L). Expression of phosphorylated MBS was also increased in AAA lesion as compared with control (Figure 8, M-O).

## Discussion

**Figure 9:**
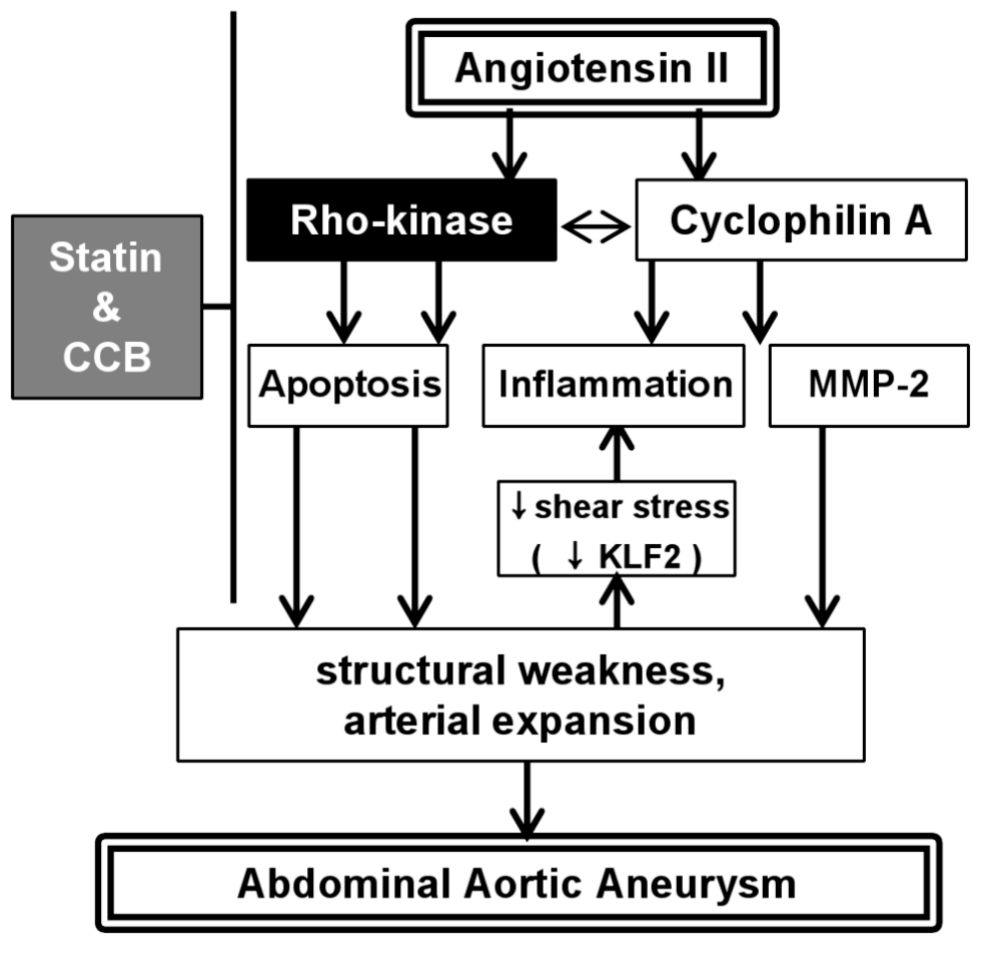
Working model of the present study. Angiotensin-II induces Rho-kinase activation as well as cyclophilin A expression associated with enhanced apoptosis, increased inflammation and activation of MMP-2, leading to structural weakness, arterial expansion and formation of abdominal aortic aneurysm. Arterial expansion induces decreased endothelial KLF-2 expression (low shear stress) causing further inflammation. In contrast, combination therapy with statin and CCB suppresses angiotensin-II-induced Rho-kinase activation with resultant inhibition of several processes and abdominal aortic aneurysm. CCB, calcium channel blocker; MMP, matrix metalloproteinase; KLF2, Krüppel-like factor2.

The major findings of the present study were that (1) the combination therapy with atorvastatin and amlodipine, but not each monotherapy, suppressed the AngII-induced AAA formation, (2) the combination therapy inhibited apoptosis, infiltration of inflammatory cells and activity of MMP in abdominal aortic tissues, (3) Rho-kinase activity was enhanced at the AAA lesion in humans and (4) experimental AAA tissues induced by Ang-II showed high Rho-kinase activity, and that it was attenuated by the combination therapy (Figure 9).

### Rho-kinase and AAA formation in humans and mice

We have previously demonstrated that Rho-kinase plays an important role in cell adhesion, migration and cytokinesis of vascular smooth muscle cells and other vascular cells [17,28]. Rho-kinase is substantially involved in the signal transduction initiated by agonists, such as AngII, leading to the development of cardiovascular disease [17]. Indeed, it was recently reported that fasudil, a selective Rho-kinase inhibitor, reduces the development of AngII-induced AAA formation [23]. These findings suggest that Rho-kinase activation is substantially involved in the pathogenesis of AAA. In the present study, we were able to demonstrate for the first time that Rho-kinase is up-regulated at the AAA lesions in humans as well as at the AngII-induced AAA lesion in mice.

### Effects of statins on AAA formation

We have recently demonstrated that pleiotropic effects of statins, especially at their clinical doses, are mediated predominantly through inhibition of the Rac1 signaling pathway, but not through Rho-kinase pathway [3]. In the present study, monotherapy with atorvastatin affected neither Rho-kinase activity nor AngII-induced maximum AAA diameter. Statin therapy reduces the progression of atherosclerosis and improves clinical outcomes in atherosclerotic vascular disease [18]. Although small clinical studies showed that statins reduced AAA growth [29] and mortality in ruptured AAA [30], others failed to observe beneficial effects of statins on AAA expansion [31,32]. At this moment, there is insufficient evidence to recommend statin use for the patients diagnosed as AAA alone (recommendation class IIb, evidence level B) [33].

### Effects of CCBs on AAA formation

There is little evidence regarding the efficacy of CCBs for the prevention of AAA growth. After endovascular aneurysm repair, CCBs enhanced sac shrinkage independent of other medications, including statins, β-blockers and ACEIs [9]. Furthermore, in the experimental study, CCB (amlodipine, 5 mg/kg/day) completely inhibited the development of AAA formation associated with significant decrease in systolic blood pressure [10]. In the present study, neither systolic blood pressure nor AAA growth was affected by the monotherapy with amlodipine (1 mg/kg/day). However, this small dose of amlodipine in addition to atorvastatin markedly reduced the AngII-induced AAA growth independent of blood pressure. Interestingly, it was recently reported that Rho-kinase activity in circulating leukocytes was greater in patients with untreated essential hypertension compared with healthy individuals and that Rho-kinase activity was significantly lower in the group treated with amlodipine compared with other groups treated with other antihypertensive agents, such as ARBs, diuretics and β-blockers [20]. Further, we have recently reported that the combination of a statin and a Rho-kinase inhibitor would exert more effective therapeutic effects compared with each monotherapy [3]. Taken together, these results suggest the combination therapy with atorvastatin and amlodipine synergistically suppresses Rho-kinase, resulting in the inhibition of AAA formation in mice in vivo.

### Inhibitory effects of the combination therapy with atorvastatin and amlodipine

Recent studies have demonstrated a potential synergistic effect of the combination with atorvastatin and amlodipine on the inhibition of vascular inflammation. In a mouse model of femoral artery injury, the combination therapy attenuated leukocyte adhesion and oxidative stress [34]. In a rat ischemic stroke model, pretreatment with atorvastatin and amlodipine ameliorated post-ischemic brain weight increase and induction of MMP-9 [35]. However, the molecular mechanisms of the beneficial effects of the combination therapy remain to be elucidated. In the present study, combination therapy with amlodipine and atorvastatin, but not each monotherapy, prevents AngII-induced AAA formation in mice. The mechanisms of how combination therapy suppressed the development of AAA formation may be involved in the inhibition of Rho-kinase with subsequent several processes. First, in the present study, apoptosis was increased at the AngII-induced AAA lesion as reported previously [23], and was markedly suppressed by the combination therapy. Second, AAA formation requires inflammation and matrix degradation. CyPA is released from vascular smooth muscle cells in a Rho-kinase-dependent manner [36]. Indeed, the Rho-kinase/CyPA pathway plays a crucial role for AAA formation by promoting inflammation and MMP activation in a mouse AAA model [15]. We have recently demonstrated that Rho-kinase inhibitor Y27632 considerably reduces CyPA secretion from vascular smooth muscle cells and that CyPA is crucial for secretion and activation of MMPs using the ApoE^-/-^Ppia^-/-^double knockout mice [15]. In the present study, the location of AngII-induced CyPA expression was mostly VSMCs which highly coincide with that of Rho-kinase (Figure 7). In addition, we found that the combination therapy suppressed Rho-kinase activity associated with CyPA expression, resulting in attenuation of inflammation and MMP-2 activity. Third, hemodynamic shear stress could influence AAA formation. Low endothelial shear stress plays an important role in the progression and formation of atherosclerotic lesion [26,37]. Recent studies suggest an involvement of low wall shear stress at the AAA site [38]. Indeed, in the present study, we were able to demonstrate for the first time that endothelial KLF2 is less expressed at the AngII-induced AAA lesion as compared with the sham group. Since KLF2 is a flow-dependent key regulator with potent anti-inflammatory and anti-thrombotic effects, suppressing monocyte attachment and thrombus formation [37], this finding suggests an importance of endothelial shear stress as a novel therapeutic target of AAA. Finally, although the combination therapy markedly reduced AngII-induced AAA lesion associated with Rho-kinase activity in this study, the detailed mechanisms of their synergistic inhibitory effects remain to be fully elucidated in future studies. Interestingly, monotherapy with clinical dose of amlodipine revealed inhibitory effect on Rho-kinase activity in human leucocytes, and 5 mg/kg/day of amlodipine completely inhibited AAA incidence in experimental aneurysm model of mice [10,20]. In the present study, amlodipine (1 mg/kg/day) slightly decreased Rho-kinase activity and apoptosis although the results did not reach statistical significance, whereas atorvastatin alone exerted no effect on that (Figures 4 and 6). Thus, the monotherapy with very low-dose alone might have lost an efficacy on Rho-kinase as well as AAA. In fact, in line with the present study, it has been recently demonstrated that monotherapy with atorvastatin (20 mg/kg/day) did not reduce AAA diameter in mice [39]. In addition, we and others have recently demonstrated that the pleiotropic effects of statins at their clinical doses (atorvastatin, 10 mg/kg/day in rats) are mediated mainly through inhibition of the Rac1 signaling pathway [3,40]. Thus, it is possible that atorvastatin in the presence of amlodipine may mediate different signaling pathway or that amlodipine with atorvastatin may exert more pronounced inhibition on Rho-kinase activity.

### Study limitations

Several limitations should be mentioned for the present study. First, although the present study was performed with the established mouse model of AngII-induced supra-renal AAA [41], AAA is prone to occur in the infra-renal abdominal aorta in humans [11]. Thus, the present findings needed to be confirmed in other models of AAA. Second, pharmacokinetics of atorvastatin and amlodipine in mice are different from those in humans [42–44]. Thus, the present findings with mice need to be confirmed in future clinical studies.

In summary, in the present study, we were able to provide the first evidence that combination therapy with atorvastatin and amlodipine, but not each monotherapy, prevents AngII-induced AAA formation in mice through inhibition of Rho-kinase/CyPA pathway with resultant inhibition of apoptosis, inflammation and MMP activation (Figure 9).

## Supporting Information

Figure S1 **Neither atorvastatin nor amlodipine affects serum lipid profile of ApoE-deficient mice.**
T-chol, total cholesterol; VLDL, very low density lipoprotein cholesterol; LDL, low density lipoprotein cholesterol; HDL, high density lipoprotein cholesterol; AngII, angiotensin II; ATOR, atorvastatin; AMLO, amlodipine; Combi, combination of atorvastatin and amlodipine. Results are expressed as mean±SEM (n=5).
(TIF)

Figure S2 **Combination therapy reduces angiotensin-II-induced elastin degeneration in the abdominal aortic aneurysm.**
**A**–**J**. Hematoxylin-eosin staining. **K**–**T**. Elastica-Masson staining. **U**. Grading of elastin degradation. L. Based on elastin degradation-grading (4 grades) keys, degradation of medial elastic lamina was statistically analyzed; grade 1, no degradation; grade 2, mild; grade 3, severe; grade 4, aortic rupture. Scale bars indicate 500 µm (**A**–**E**, **K**–**O**) and 100 µm (**F**–**J**, **P**–**T**).
HE, Hematoxylin-Eosin; EM, Elastica-Masson staining; AngII, angiotensin II; ATOR, atorvastatin; AMLO, amlodipine; Combi, combination of atorvastatin and amlodipine. Results are expressed as mean±SEM (n=7-10).
(TIF)

Figure S3 **Combination therapy improves angiotensin-II-induced down-regulation of endothelial Krüppel-like factor2 (**KLF2**) expression.**
**A**. A representative photograph of color Doppler ultrasonography showing turbulent flow at the AngII-induced abdominal aortic aneurysm lesion in ApoE^-/-^ mice. White arrowheads indicate the location of turbulent flow.
**B**. Representative photographs of immunohistochemistry of KLF2. Black arrowheads indicate KLF2-positive cells. Scale bars indicate 100 µm.
KLF2, Krüppel-like factor2; AngII, angiotensin II; ATOR, atorvastatin; AMLO, amlodipine; Combi, combination of atorvastatin and amlodipine.
(TIF)

Table S1 **Blood pressure.**
(DOC)

Table S2 **Sample numbers in the experiments.**
(DOC)

Text S1 **Supplementary methods.**
(DOC)
