# Supplementary figures and images for: Combination Therapy with Atorvastatin and Amlodipine Suppresses Angiotensin II-Induced Aortic Aneurysm Formation
Source: PLoS One. 2013 Aug 13;8(8):e72558. doi: 10.1371/journal.pone.0072558 (PMC3742630; doi:10.1371/journal.pone.0072558)

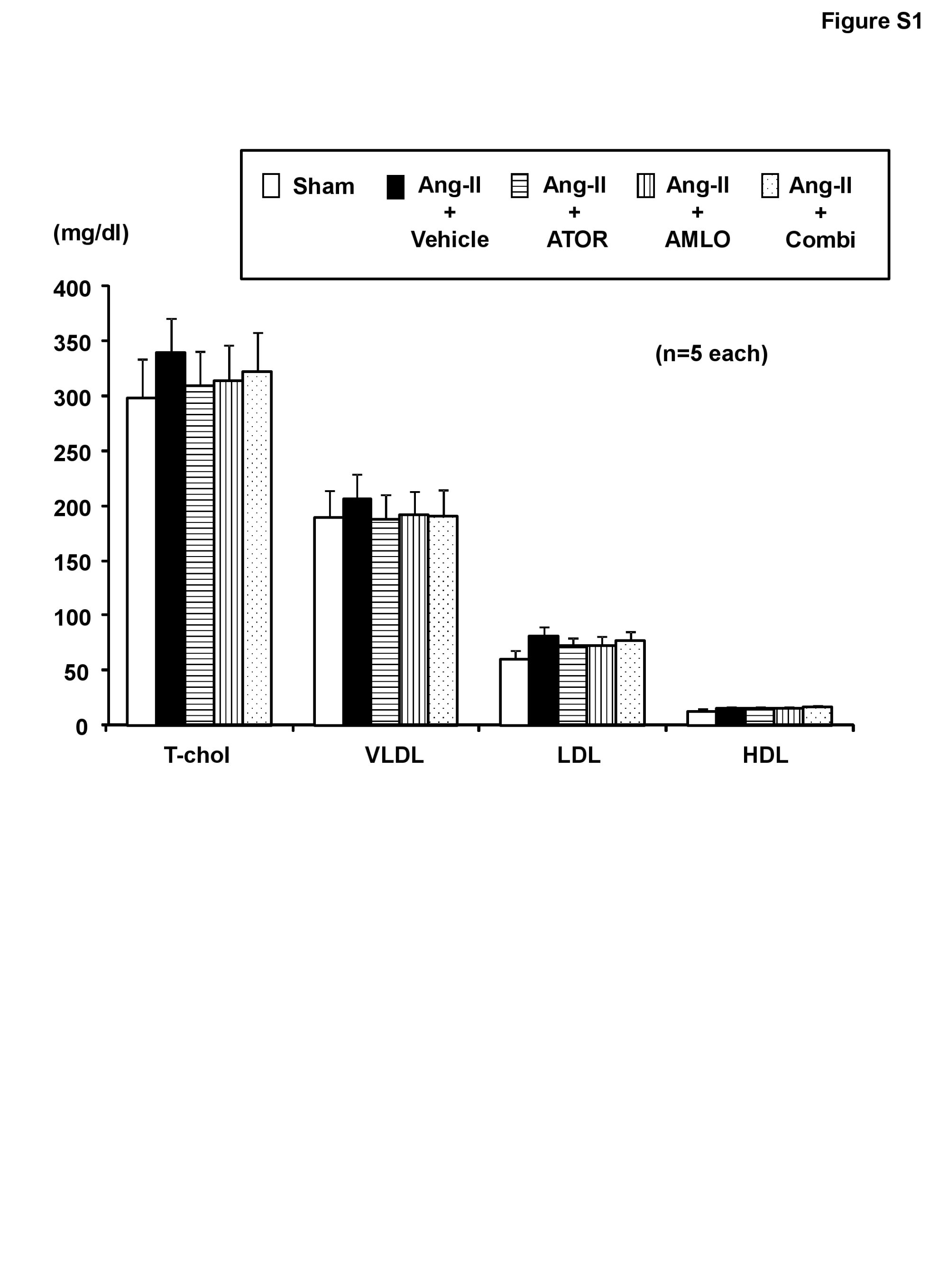

Supplement: Figure S1 — Neither atorvastatin nor amlodipine affects serum lipid profile of ApoE-deficient mice. T-chol, total cholesterol; VLDL, very low density lipoprotein cholesterol; LDL, low density lipoprotein cholesterol; HDL, high density lipoprotein cholesterol; AngII, angiotensin II; ATOR, atorvastatin; AMLO, amlodipine; Combi, combination of atorvastatin and amlodipine. Results are expressed as mean±SEM (n=5). (TIF) [file pone.0072558.s001.tif]

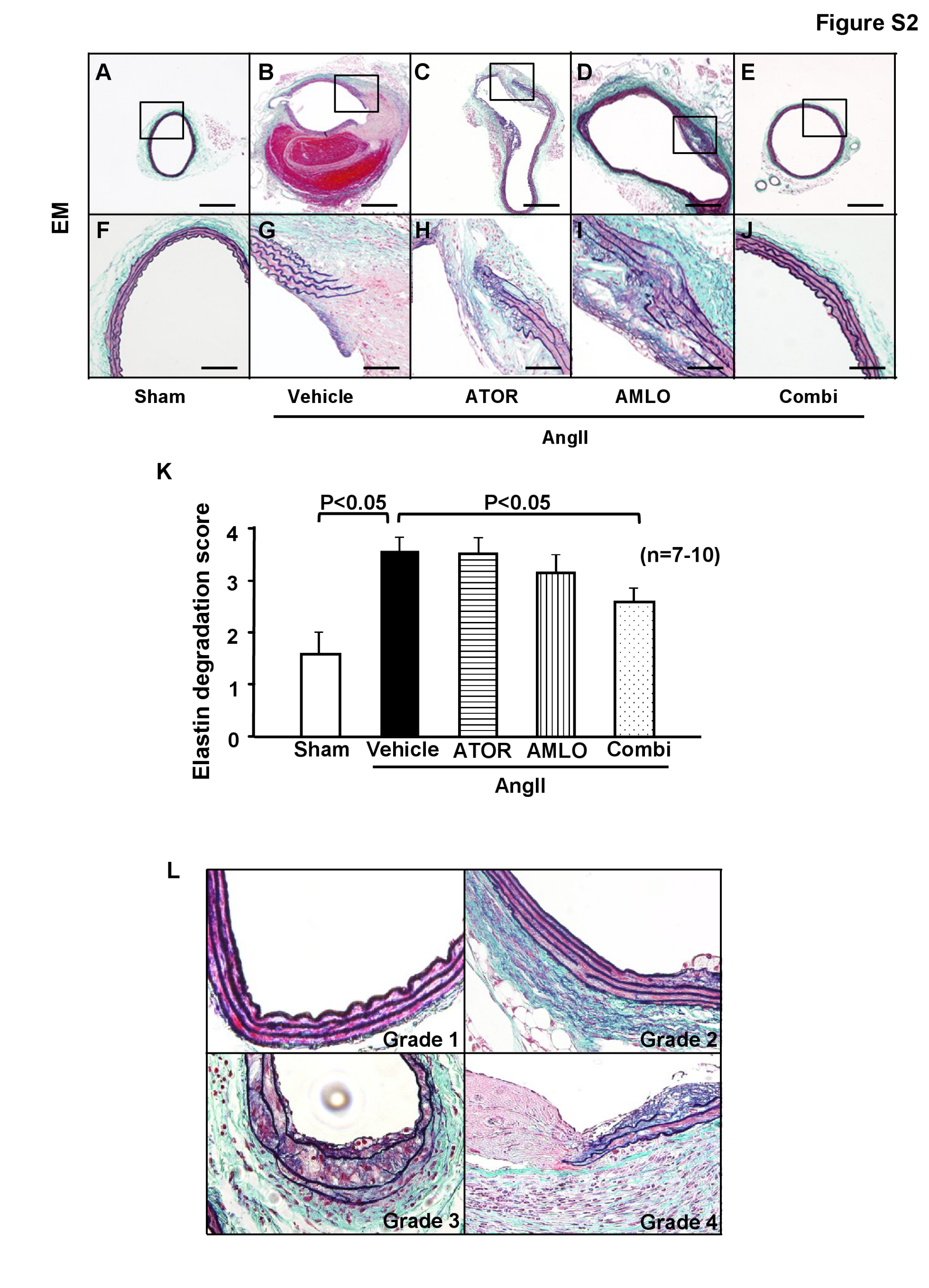

Supplement: Figure S2 — Combination therapy reduces angiotensin-II-induced elastin degeneration in the abdominal aortic aneurysm. A–J. Hematoxylin-eosin staining. K–T. Elastica-Masson staining. U. Grading of elastin degradation. L. Based on elastin degradation-grading (4 grades) keys, degradation of medial elastic lamina was statistically analyzed; grade 1, no degradation; grade 2, mild; grade 3, severe; grade 4, aortic rupture. Scale bars indicate 500 µm (A–E, K–O) and 100 µm (F–J, P–T). HE, Hematoxylin-Eosin; EM, Elastica-Masson staining; AngII, angiotensin II; ATOR, atorvastatin; AMLO, amlodipine; Combi, combination of atorvastatin and amlodipine. Results are expressed as mean±SEM (n=7-10). (TIF) [file pone.0072558.s002.tif]

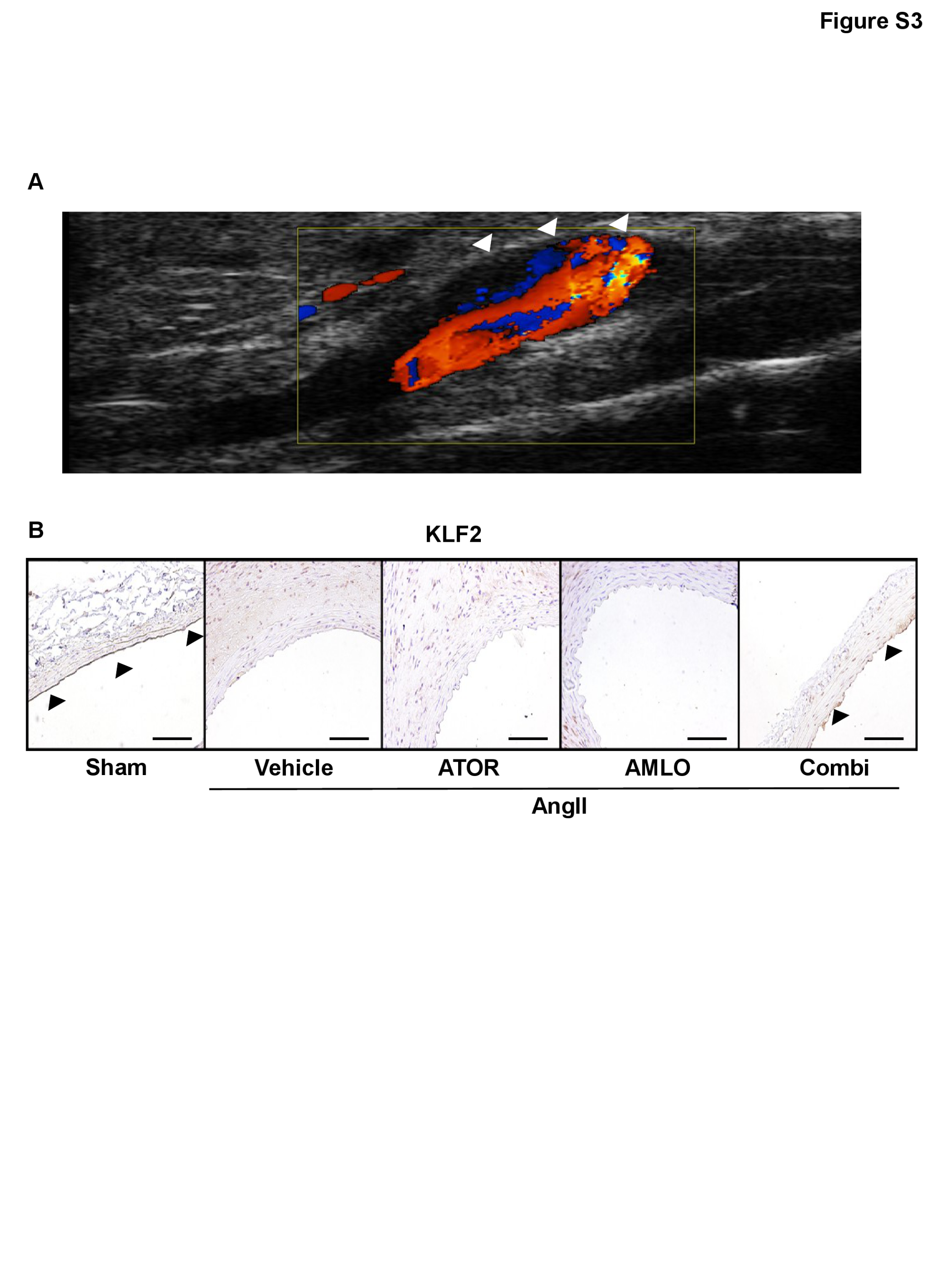

Supplement: Figure S3 — Combination therapy improves angiotensin-II-induced down-regulation of endothelial Krüppel-like factor2 (KLF2) expression. A. A representative photograph of color Doppler ultrasonography showing turbulent flow at the AngII-induced abdominal aortic aneurysm lesion in ApoE-/- mice. White arrowheads indicate the location of turbulent flow. B. Representative photographs of immunohistochemistry of KLF2. Black arrowheads indicate KLF2-positive cells. Scale bars indicate 100 µm. KLF2, Krüppel-like factor2; AngII, angiotensin II; ATOR, atorvastatin; AMLO, amlodipine; Combi, combination of atorvastatin and amlodipine. (TIF) [file pone.0072558.s003.tif]
