# Supplementary material for: Combination Therapy with Atorvastatin and Amlodipine Suppresses Angiotensin II-Induced Aortic Aneurysm Formation
Source: PLoS One. 2013 Aug 13;8(8):e72558. doi: 10.1371/journal.pone.0072558 (PMC3742630; doi:10.1371/journal.pone.0072558)
Supplement: Table S1 — Blood pressure. (DOC) [file pone.0072558.s004.doc]

**Supplementary Table 1**

**Blood pressure**

|  | -1W | 0W | 1W | 2W | 3W | 4W |
| --- | --- | --- | --- | --- | --- | --- |
| Sham | 104.85±3.79 | 100.68±3.67 | 101.52±7.19 | 103.81±7.00 | 100.17±8.54 | 102.79±8.49 |
| AngII | 107.48±2.30 | 108.75±2.50 | 140.24±4.05^†^ | 141.43±4.04^†^ | 146.60±4.93^†^ | 143.72±5.02^†^ |
| ATOR | 105.91±2.19 | 107.90±2.12 | 146.76±4.15^†^ | 147.30±4.04^†^ | 149.36±4.93^†^ | 150.19±4.90^†^ |
| AMLO | 107.37±2.19 | 107.05±2.12 | 143.46±4.36^†^ | 147.68±4.25^†^ | 149.24±5.18^†^ | 143.17±5.30^†^ |
| Combi | 105.98±2.19 | 109.85±2.12 | 142.18±4.25^†^ | 141.18±4.14^†^ | 148.27±5.05^†^ | 145.93±5.02^†^ |

(mmHg) (n=18-21)

^†^P< 0.01 vs. saline infusion or before treatment. Results are mean ± SEM
