# Supplementary material for: Combination Therapy with Atorvastatin and Amlodipine Suppresses Angiotensin II-Induced Aortic Aneurysm Formation
Source: PLoS One. 2013 Aug 13;8(8):e72558. doi: 10.1371/journal.pone.0072558 (PMC3742630; doi:10.1371/journal.pone.0072558)
Supplement: Table S2 — Sample numbers in the experiments. (DOC) [file pone.0072558.s005.doc]

**Supplementary Table 2**

**Sample numbers in the experiments**

|  | Morphology | Zymography | Western blot | Sudden death(rupture, etiology unknown) | Total |
| --- | --- | --- | --- | --- | --- |
| Sham | 7 | 8 | 7 | 0 (0,0) | 22 |
| AngII | 20 | 8 | 10 | 6 (5,1) | 44 |
| ATOR | 21 | 8 | 8 | 2 (2,0) | 39 |
| AMLO | 18 | 8 | 7 | 4 (2,2) | 37 |
| Combi | 20 | 8 | 9 | 3 (2,1) | 40 |
