# Supplementary material for: Combination Therapy with Atorvastatin and Amlodipine Suppresses Angiotensin II-Induced Aortic Aneurysm Formation
Source: PLoS One. 2013 Aug 13;8(8):e72558. doi: 10.1371/journal.pone.0072558 (PMC3742630; doi:10.1371/journal.pone.0072558)
Supplement: Text S1 — Supplementary methods. (DOC) [file pone.0072558.s006.doc]

**Supplementary methods**

**Lipid analysis**

Plasma samples were obtained from the mice at the time of sacrifice following 28 days of AngII infusion. The animals were anesthetized with 3.0% isoflurane, and blood was collected from the inferior vena cava using a plastic microsyringe. The plasma levels of lipids were analyzed with a high-performance liquid chromatography system by Skylight Biotech [1].

**Blood pressure measurements**

Systolic blood pressure was measured at room temperature by a tail cuff method using an MK2000 blood pressure monitor (Muromachi Kikai, Tokyo, Japan), under conscious conditions. Mice were acclimated to the procedures of blood pressure measurement for a week preceding actual data collection. Systolic blood pressures were measured 8-days before pump implantation to record baseline blood pressures, 1-day before pump implantation, and repeated weekly during AngII infusion.

**Histology and immunohistochemistry**

Morphological and immunohistochemical analyses were performed as previously described [2].　 For morphometric studies, the mice were anaesthetized with 3.0% isoflurane followed by blood collection and perfusion via cardiac puncture with 10% formalin. The depth of general anaesthesia was assessed by pinching tail of the mouse. Any reaction from the mouse indicated that the anaesthesia was too light and that higher concentration of isoflurane should be given. The entire aorta from the ascending aorta to the iliac bifurcation were exposed and placed in 10% formalin for 24 h to complete the fixation. Fixed aortic tissues were then embedded in paraffin, and then cut into 3-µm sections for morphological and immunohistochemical analysis. Elastica-Masson staining was performed and grading of elastin degradation were performed based on the previous study [3]; grade 1, no degradation; grade 2, mild; grade 3, severe; grade 4, aortic rupture. Each aortic tissue was scored in 7 fields and the average was used as an individual value. Immunohistochemistry was performed on histological sections of formalin-fixed paraffin-embedded tissue samples using rabbit anti-mouse Krüppel-like factor 2 (KLF2, Bioss, Woburn, MA, USA) antibody.

**Ultrasonography**

Each mouse was anesthetized using 1.5% isoflurene during ultrasonography. Ultrasonography was performed by the sophisticated high-resolution ultrasound imaging system (Vevo 2100, VisualSonics, Toronto, Canada) with [color Doppler mode](http://www.visualsonics.com/info.php?pid=618) for detection of blood vessels including flow directional information, providing a visual overview of flow within the vessel. Flow direction can be delineated by red and blue color spectrums.

**Supplementary References**

1. Nakajima S, Ohashi J, Sawada A, Noda K, Fukumoto Y, Shimokawa H. Essential role of bone marrow for microvascular endothelial and metabolic functions in mice. *Circ Res* 2012;**111**:87-96.

2. Matsumoto Y, Adams V, Jacob S, Mangner N, Schuler G, Linke A. Regular exercise training prevents aortic valve disease in low-density lipoprotein-receptor-deficient mice. *Circulation* 2010;**121**:759-767.

3. Satoh K, Nigro P, Matoba T, O'Dell MR, Cui Z, Shi X*, et al.* Cyclophilin A enhances vascular oxidative stress and the development of angiotensin II-induced aortic aneurysms. *Nat Med* 2009;**15**:649-656.
